# Rhabdomyolysis and Neurological Manifestation With Progressive Weakness in a Young Adult: A Rare Extrapulmonary Presentation of Mycoplasma Pneumoniae

**DOI:** 10.7759/cureus.20552

**Published:** 2021-12-20

**Authors:** Ojbindra KC, Punya H Dahal, Manisha Koirala, Afua D NtemMensah

**Affiliations:** 1 Hospital Medicine, Faith Regional Health Services, Norfolk, USA; 2 Infectious Disease, Faith Regional Health Services, Norfolk, USA

**Keywords:** immune globulin, pneumonia, mycoplasma, neuropathy, weakness, macrolide resistance, neurological manifestation, rhabdomyolysis

## Abstract

*Mycoplasma pneumoniae (M. pneumoniae) *is a common cause of community-acquired pneumonia. It has been associated with many extrapulmonary manifestations that can present even in the absence of pulmonary signs and symptoms. Rhabdomyolysis and central nervous system (CNS) manifestations are rare extrapulmonary manifestations. These are infrequently reported in adults. We present a case of a healthy 32-year-old male who initially presented with signs and symptoms of community-acquired pneumonia and was treated with antibiotics. However, he continued to have generalized malaise, night sweats, diffuse joint pain, and myalgias and was subsequently noted to have rhabdomyolysis with elevated creatine kinase (CK) and myoglobin levels. Rhabdomyolysis was attributed to *M. pneumoniae* based on the recent history of upper respiratory tract infection and *M. pneumoniae* immunoglobulin M (IgM) serology positivity along with high *M. pneumoniae* IgG titer. The other causes of rhabdomyolysis were diligently excluded based on patient history and laboratory and clinical data. This immune-mediated rhabdomyolysis improved with intravenous hydration, doxycycline, and prednisone therapy. However, the patient developed progressive weakness with neuropathy, which required treatment with intravenous immune globulin (IVIG). This case highlights the need to maintain a high index of suspicion for rare extrapulmonary manifestations of mycoplasma infection, which could be life-threatening or cause significant morbidity; and in cases of severe extrapulmonary manifestations, the appropriate use of immunosuppressive/immunomodulatory therapy may lead to a better outcome.

## Introduction

*Mycoplasma pneumoniae (M. pneumoniae) *causes acute respiratory illness, which ranges in severity from mild upper respiratory tract infection to pneumonia. It is associated with an annual incidence rate that amounts to approximately 1% of the United States population. It can affect any age group but is more common in children aged more than five years and young adults aged less than 40 years [1,2]. The incubation period is about two to three weeks, and patients can be completely asymptomatic or present with clinical symptoms of headache, malaise, fever, chills, and cough; 3-10% of infected people can develop pneumonia [3]. An extrapulmonary disease that can include hematological, dermatological, cardiac, gastrointestinal, neurological, and musculoskeletal manifestations can occur in about 5-10% of patients infected with mycoplasma and can appear before, during, after, or in the absence of pulmonary signs [3,4]. Polyarthralgia and myalgia are common musculoskeletal manifestations but rhabdomyolysis is rare; most cases involve pediatric patients and only a handful of cases have been reported in healthy adults [5-14]. Central nervous system (CNS) manifestations can present as meningitis, peripheral neuropathy, transverse myelitis, Guillain-Barré syndrome, acute disseminated encephalomyelitis, cranial nerve palsies, or cerebellar ataxia and occur in approximately 0.1% of patients with *M. pneumoniae* infection [15]. In this report, we present a rare case of rhabdomyolysis followed by neurological manifestation with progressive weakness secondary to *M. pneumoniae *infection in a young adult.

## Case presentation

A 32-year-old-male with no significant past medical history presented to the emergency room with complaints of chills, cough, shortness of breath, chest congestion, and generalized malaise for two days. He denied fever, chest pain, nausea, vomiting, diarrhea, dysuria, abdominal pain, and any sick contact or travel history. He was not taking any prescribed or over-the-counter medications. Initial vitals revealed a temperature of 98.2 °F, heart rate of 100 beats/minute, blood pressure of 140/85 mmHg, and respiratory rate of 18 breaths/minute with 98% oxygen saturation on room air. Physical examination was significant for reduced breath sounds bilaterally with mild expiratory wheezing. The rest of the physical examination was unremarkable. Initial laboratory workup was significant for a leukocyte count of 22.6 x 10^3^/µl with 83.9% neutrophils; the rest of the laboratory findings were unremarkable (Table 1).

**Table 1:** Initial laboratory studies during first, second, and third hospitalizations BUN: blood urea nitrogen; CK: creatine kinase; CRP: c-reactive protein; ESR: erythrocyte sedimentation rate; IgM: immunoglobulin M

Variables	Reference range	Laboratory studies at first admission with pneumonia	Laboratory studies at second admission with rhabdomyolysis	Laboratory studies at third admission with neuropathy
White blood cell count	4.0-10.0 X10^3^/µl	22.6 x 10^3^/µl	17.5 x 10^3^/µl	15.8 x 10^3^/µl
Hemoglobin	13.5-17.0 gm/dl	16.9 gm/dl	17.1 gm/dl	16.3 gm/dl
Platelets	150-450 x10^3^/µl	275 x 10^3^/µl	278 x 10^3^/µl	259 x 10^3^/µl
Sodium	134-146 mEq/L	141 mEq/L	140 mEq/L	143 mEq/L
Potassium	3.5-5.3 mEq/L	3.9 mEq/L	4.2 mEq/L	3.8 mEq/L
Chloride	98-110 mEq/L	106 mEq/L	104 mEq/L	107 mEq/L
Bicarbonate	21-30 mM/L	21 mM/L	11 mM/L	24 mM/L
BUN	7.0-25 mg/dl	11 mg/dl	25 mg/dl	11 mg/dl
Creatinine	0.7-1.30 mg/dl	0.92 mg/dl	0.92 mg/dl	0.79 mg/dl
Glucose	65-95 mg/dl	86 mg/dl	82 mg/dl	81 mg/dl
Procalcitonin	<0.10 ng/ml	0.02 ng/ml	0.03 ng/ml	0.03 ng/ml
CK levels	40-250 U/L	Not checked	2537 U/L	157 U/L
Myoglobin	28-72 ng/ml	Not checked	1258 ng/ml	Not checked
ESR	0-15 mm/hr	Not checked	4 mm/hr	6 mm/hr
CRP	<0.8 mg/dl	Not checked	0.04 mg/dl	0.04 mg/dl
*M. pneumoniae *IgM	Non-reactive	Not checked	Reactive	Reactive
*M. pneumoniae *IgG	<0.09 U/L	Not checked	Not checked	0.21 U/L

**Figure 1:**
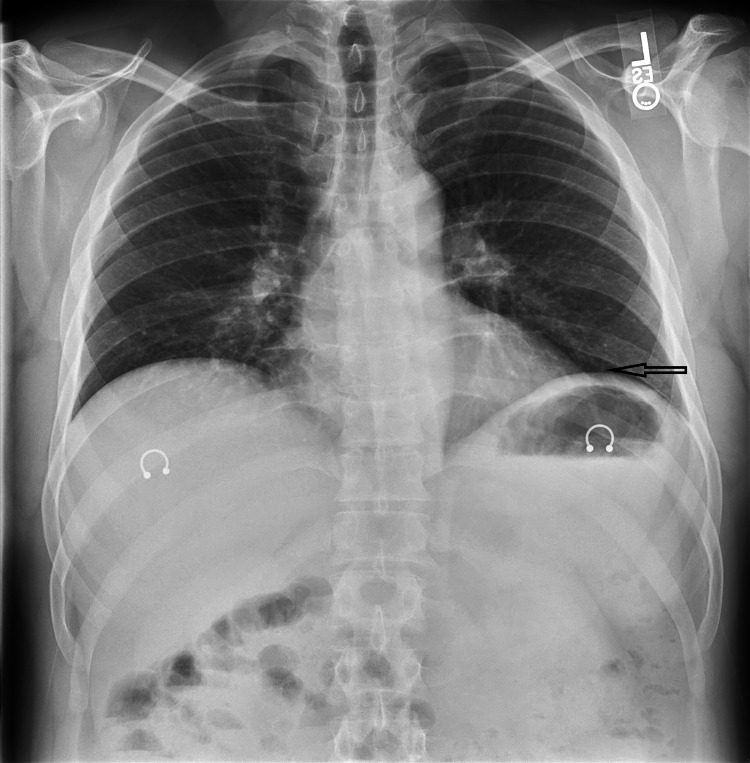
Chest X-ray showing faint infiltrate on the left lung base (arrow)

The chest X-ray revealed a faint infiltrate on the left lung base (Figure 1).

Additional workup with respiratory pathogen panel with nucleic acid amplification test (NAAT), which includes influenza A, influenza B, parainfluenza, human coronavirus, human metapneumovirus, *Bordetella pertussis*, *Chlamydia pneumoniae*, and *M. pneumoniae, *were negative. Urine *Streptococcus* and *Legionella* antigen were negative as well. The patient was hospitalized and treated with albuterol nebulization, intravenous methylprednisolone, and intravenous ceftriaxone, and oral azithromycin. His leukocytosis trended down and procalcitonin remained negative along with blood cultures for 48 hours and he was discharged home on azithromycin, steroid taper, and albuterol inhaler for wheezing as needed.

**Figure 2:**
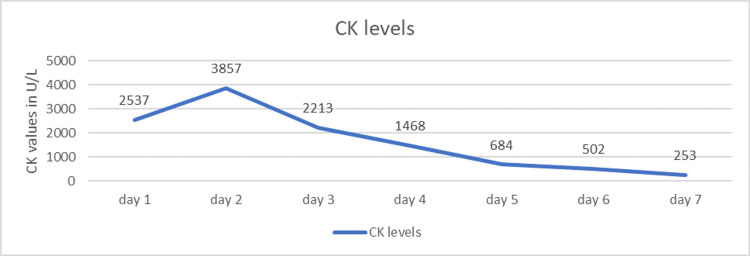
Creatine kinase (CK) level trend during hospitalization

He followed up with his primary care physician (PCP) after one week of discharge and reported that his cough, chest congestion, and shortness of breath had resolved but stated that he continued to experience excessive fatigue with intermittent subjective fever, night sweats with diffuse joint pain, and myalgia. His vital signs and physical examination were unremarkable. His repeat laboratory workup was significant for persistent leukocytosis of 17.5 x 10^3^/µl, elevated creatine kinase (CK) levels of 2537 U/L, and elevated myoglobin level of 1258 U/L. The rest of the laboratory workup was unremarkable as summarized in Table 1. Additional workup including serum cortisol, thyroid-stimulating hormone (TSH), complements levels (C3, C4, CH50), antinuclear antibodies (ANA), anti-neutrophilic cytoplasmic autoantibody (ANCA), and anti-cyclic citrullinated peptides (anti-CCP) were unremarkable; however, *M. pneumoniae* IgM was reactive. He was hospitalized and started on intravenous normal saline at 200 ml/hour and CK levels were monitored closely. Since he had been previously treated with azithromycin, doxycycline was started for positive *M. pneumoniae* IgM given the possibility of mycoplasma resistance to macrolides. Despite aggressive intravenous hydration and doxycycline, his CK level trended upwards and he continued to have generalized muscle soreness; he was started on prednisone 1 mg/kg dose for immune-mediated rhabdomyolysis. His CK level gradually trended down along with improvement in his myalgia (Figure 2).

He was discharged home on the seventh day of admission with a CK level of 253 U/L with oral prednisone taper.

Within 24 hours of discharge, he started to have bilateral lower extremity weakness and suffered multiple falls at home, and was readmitted the next day. On readmission, his vitals were unremarkable; however, physical examination was significant for quadriparesis with a muscle power of 4/5 (Table 2), with intact deep tendon reflex, and decreased sensation to pinprick in bilateral lower extremities below his knees and bilateral upper extremity below his elbows.

**Table 2:** The Medical Research Council's grading system for muscle strength

Grade	Characteristics
Grade 5	Muscle contracts normally against full resistance
Grade 4	Muscle strength is reduced, but contraction can still move joint against resistance
Grade 3	Joint can be moved only against gravity with the examiner’s resistance completely removed
Grade 2	Muscle can move only if the resistance of gravity is removed
Grade 1	Only a trace or flicker of movement is seen or felt in muscles or fasciculation is observed
Grade 0	No movement is observed

His laboratory results are summarized in Table 1, and his CK level was within normal limits. Imaging studies with MRI brain and spine with and without contrast were unremarkable. A lumbar puncture was done, which was also unremarkable (Table 3).

**Table 3:** Cerebrospinal fluid studies after lumbar puncture CSF: cerebrospinal fluid; PCR: polymerase chain reaction; RBC: red blood cells

Variables	Reference range	Observed values
RBC	0 RBC/cmm	<1000
Nucleated cells	0-10/cmm	<20
Lymphocytes	40-80%	40%
Neutrophils	0-6%	36%
Monocytes	15-45%	24%
Glucose	55-80 mg/dl	55 mg/dl
Protein	14-45 mg/dl	25 mg/dl
CSF mycoplasma by PCR	-	Not detected
Neuromuscular antibody CSF anti-GQ1b, anti-GM1	-	Not detected

CNS meningitis/encephalitis panel was unremarkable. Additional workup including HIV, syphilis, herpes simplex virus, human T lymphotropic virus, coxsackievirus, and West Nile virus was unremarkable. Lyme disease antibodies, *Coccidioides* antibodies, Blastomyces antibodies, toxoplasma IgG antibodies, *Anaplasma* serum antigen, and hepatitis C antibodies were also unremarkable. The patient was continued on oral prednisone 1 mg/kg. However, the next day, he started to have a progressive weakness with decreasing muscle power of 3/5 (Table 2) in all four extremities along with the progression of numbness with decreased pinprick sensation from lower extremity to the thoracic level of T-10; his deep tendon reflexes were intact. He was started on intravenous immune globulin (IVIG) infusion at 0.4 mg/kg and monitored closely in the ICU. His weakness and numbness gradually improved with IVIG, which was given for five days. He worked with physical and occupational therapy and was able to ambulate with the help of a walker on the day of discharge. He followed up with neurology outpatient and electromyography (EMG) was done after two weeks, which was unremarkable. He recovered completely after a total of six weeks of outpatient physical therapy.

## Discussion

Rhabdomyolysis involves the breakdown of skeletal muscles resulting in the release of intracellular components, including myoglobin, CK, and other intracellular electrolytes in circulation. Clinically, it can be asymptomatic or present with myalgia, muscle swelling, muscle tenderness, or passage of tea-colored urine. The diagnosis is usually based on myalgia or urine analysis (UA) that tests positive for blood in the absence of red blood cells on microscopy. Serum CK level greater than five times the upper limit of the normal range is the hallmark of rhabdomyolysis and is sufficient to establish the diagnosis [16]. It is usually managed with aggressive intravenous hydration to prevent myoglobin-induced renal failure, which could develop in about 50% of patients with rhabdomyolysis, along with treatment of the causative factors [4]. There are various factors that can cause it, which include trauma, extreme exertion, heatstroke, metabolic myopathy, hypothyroidism, electrolytes disorders (hypokalemia, hypophosphatemia), medication side effects, and certain infections [16]. Infections account for less than 5% of all causes of rhabdomyolysis and include influenza A, influenza B, coxsackievirus, cytomegalovirus, Epstein-Barr virus (EBV), varicella-zoster virus, parainfluenza virus, adenovirus, echovirus, HIV, *Legionella,* *Streptococcus, Salmonella,* *Escherichia coli*, *Clostridium perfringens*, chlamydia, and *M. pneumoniae* [17].

Rhabdomyolysis triggered by *M. pneumoniae* is a rare extrapulmonary manifestation and has been infrequently reported in healthy adults [5-9]. The pathogenesis of rhabdomyolysis is not completely understood and the proposed mechanism includes immune-mediated reaction or direct invasion of affected muscles by the organism [16]. CNS manifestation is another rare extrapulmonary manifestation and occurs in approximately 0.1% of patients with mycoplasma infection [15]. Encephalitis is a common CNS manifestation but can present with meningitis, peripheral neuropathy, Guillain-Barré syndrome, cranial nerve palsies, transverse myelitis, and cerebellar ataxia. The prognosis varies with the type and extent of CNS involvement but can be poor [15].

Diagnosis of mycoplasma infection usually depends on polymerase chain reaction (PCR) gene amplification technique or serological test as the organism is fastidious and difficult to culture. PCR is typically done when it is available but, in most cases, serology for *M. pneumoniae* IgM is performed. In one study, the overall sensitivity of PCR was 40% compared to 40% and 35% for IgM and IgG/IgM assay respectively [18]. The sensitivity of PCR declines with the passage of time since infection. IgM titer rises earlier than IgG (approximately seven-nine days into infection); the gold standard for serologic diagnosis requires a four-fold rise in IgG titers both during acute infection and during the recovery phase (approximately four weeks later), which is generally impractical. Hence, a single high IgM titer can be used to make a presumptive diagnosis [19].

Antibiotic therapy with macrolides, fluoroquinolones, or tetracyclines is the mainstay of treatment for non-respiratory mycoplasma presentation. However, the use of immunosuppressive agents or immunomodulatory therapy including glucocorticoids, IVIG, and plasmapheresis is warranted in some extrapulmonary diseases, especially CNS manifestations or severe hematological involvement [4,15]. Macrolide resistance has been increasing in frequency and it has become important to consider macrolide resistance when selecting antibiotic treatment [20].

In our case, rhabdomyolysis was attributed to *M. pneumoniae* based on the recent history of upper respiratory tract infection and* M. pneumoniae *IgM serology positivity along with high *M. pneumoniae* IgG titer, and other causes were diligently excluded based on patient history and laboratory and clinical data. On initial presentation, the patient was adequately treated for community-acquired pneumonia with atypical coverage with azithromycin. However, he continued to have active infection with positive *M. pneumoniae* IgM, which pointed towards macrolide resistance. Subsequently, he developed rhabdomyolysis followed by progressive weakness and numbness with neuropathy, which is an unusual and rare extrapulmonary manifestation of *M. pneumoniae* infection, and required treatment with intravenous hydration, high-dose prednisone, and IVIG.

## Conclusions

Extrapulmonary manifestations of mycoplasma infection can cause significant morbidity and even be life-threatening. This case highlights the need to maintain a high index of suspicion for rare extrapulmonary manifestations such as rhabdomyolysis and progressive neuropathy. The use of appropriate antibiotics against mycoplasma infection with consideration for macrolide resistance along with immunosuppressive or immunomodulatory therapy for severe extrapulmonary manifestations may lead to a better outcome as was evident in our case.
